# Soil Moisture Variations in Response to Precipitation Across Different Vegetation Types on the Northeastern Qinghai-Tibet Plateau

**DOI:** 10.3389/fpls.2022.854152

**Published:** 2022-04-06

**Authors:** Licong Dai, Ruiyu Fu, Xiaowei Guo, Yangong Du, Fawei Zhang, Guangmin Cao

**Affiliations:** ^1^College of Ecology and Environment, Hainan University, Haikou, China; ^2^Qinghai Provincial Key Laboratory of Restoration Ecology for Cold Regions, Northwest Institute of Plateau Biology, Chinese Academy of Sciences, Xining, China; ^3^Key Laboratory of Adaptation and Evolution of Plateau Biota, Northwest Institute of Plateau Biology, Chinese Academy of Sciences, Xining, China; ^4^Hainan Academy of Forestry, Haikou, China

**Keywords:** soil moisture, Qinghai-Tibet Plateau, precipitation infiltration, temporal variations, vegetation type

## Abstract

An understanding of soil moisture conditions is crucial for hydrological modeling and hydrological processes. However, few studies have compared the differences between the dynamics of soil moisture content and soil moisture response to precipitation infiltration under different types of vegetation on the Qinghai-Tibet Plateau (QTP). In this study, a soil moisture sensor was used for continuous volumetric soil moisture measurements during 2015 and 2016, with the aim of exploring variations in soil moisture and its response to precipitation infiltration across two vegetation types (alpine meadow and alpine shrub). Our results showed that temporal variations in soil moisture at the surface (0–20 cm) and middle soil layers (40–60 cm) were consistent with precipitation patterns for both vegetation types. However, there was a clear lag in the soil moisture response to precipitation for the deep soil layers (80–100 cm). Soil moisture content was found to be significantly positively related to precipitation and negatively related to air temperature. Aboveground biomass was significantly negatively associated with the surface soil moisture content (0–20 cm) during the growing season. Statistically significant differences were observed between the soil water content of the surface, middle, and deep soil layers for the two vegetation types (*p* < 0.05). Soil moisture (19.81%) in the surface soil layer was significantly lower than that in the deep soil layer (24.75%) for alpine shrubs, and the opposite trend was observed for alpine meadows. The maximum infiltration depth of alpine shrubs was greater than that of alpine meadows under extremely high-precipitation events, which indicates that alpine shrubs might be less susceptible to surface runoff under extreme precipitation events. Furthermore, low precipitation amounts did not affect precipitation infiltration for either vegetation type, whereas the infiltration depth increased with precipitation for both vegetation types. Our results suggest that a series of small precipitation events may not have the same effect on soil moisture as a single large precipitation event that produces the equivalent total rainfall.

## Introduction

Soil moisture is a key component of the hydrological cycle of terrestrial ecosystems (Schwinning and Sala, 2004; Song et al., 2007) and provides an important link between soil properties and plants, which play critical roles in vegetation restoration and hydrological processes, such as infiltration, evaporation, and the uptake of groundwater and root water (Puri et al., 2011). However, soil moisture is often heterogeneous in both temporal and spatial dimensions, even at small scales (Gómez-Plaza et al., 2000), and is affected by factors such as vegetation (Vivoni et al., 2010), topography (Svetlitchnyi et al., 2003; Yang et al., 2012), soil type (Tomer and Anderson, 1995), and land use (Biro et al., 2013). Characterizing soil moisture and its spatial and temporal variability is, therefore, a key focus area for research into water resource management and hydrological processes.

Many studies have investigated the dynamics and magnitude of temporal and spatial changes to soil moisture distribution driven by land-use change (Gómez-Plaza et al., 2000; Chen et al., 2007), and previous studies have focused on understanding the dominant influences on soil moisture variability at a catchment scale and at larger scales (Deng et al., 2016; Mei et al., 2018). For example, Niu et al. (2015) analyzed soil moisture conditions under different land uses in an arid region of Horqin Sandy Land in northern China and found that soil moisture was sensitive to differences in land use. They also showed that temporal variability in soil moisture did not always agree with rainfall events. Other studies have suggested that vegetation type and precipitation characteristics have the strongest influence on soil moisture across soil profiles (Chen et al., 2007; Duan et al., 2016). Moreover, surface soil moisture is greatly affected by the amount and intensity of precipitation, whereas the moisture content of deeper soil layers is mainly determined by vegetation type (Cheng et al., 2020). For instance, Yao et al. (2013) found that surface soil moisture is more sensitive to precipitation than soil moisture in deeper soil layers.

In addition to different land uses and precipitation patterns, soil moisture is also affected by vegetation cover and topography. A previous study found that vegetation cover is negatively related to soil moisture because the plant canopy intercepts rainfall, and plant growth can consume soil water (Yang et al., 2018). In comparison with soil moisture, slope position and aspect may affect shallow soil moisture more than deep layers, whereas slope gradient considerably affects both shallow and deep soil moisture (Yang et al., 2012). Furthermore, precipitation patterns and vegetation cover also affect soil infiltration processes, as well as antecedent soil water content (Guo et al., 2015; Dai et al., 2019a) and soil properties (Neris et al., 2012). Several studies have shown that soil infiltration is significantly affected by precipitation intensity and that infiltration depth increases with precipitation amount (Yao et al., 2013; Niu et al., 2015). Overall, the previous research has mostly been conducted in temperate, semiarid, and arid regions such as the Loess Plateau and Inner Mongolia in China (Yao et al., 2013; Cheng et al., 2020). The dynamics that control soil moisture in alpine ecosystems have received less attention, and the effects of different vegetation types have been neglected. The assessment of soil moisture dynamics for different vegetation types in an alpine ecosystem can provide much-needed insights into the effective use of water resources in alpine environments.

The 2.5 million km^2^ of alpine grassland, which covers nearly 60% of the Qinghai-Tibet Plateau (QTP), is the largest area of alpine grassland in the world (Dong et al., 2020). Streams in the surrounding regions are important water resources for China and Southeast Asia (Dai et al., 2019a) and most originate in the QTP. The previous studies in this region have focused on the effects of alpine meadow degradation on soil water retention (Zeng et al., 2013; Pan et al., 2016; Dai et al., 2020a,b), rather than assessing soil moisture dynamics and responses to precipitation infiltration under different vegetation types. In this study, we aimed to describe how soil moisture responds to precipitation across different vegetation types in the QTP. The objectives were to explore (1) the dynamics that control soil moisture for two types of vegetation, (2) the response of soil moisture to different precipitation events under different vegetation types, and (3) differences in the vertical soil moisture distribution under different vegetation types.

## Materials and Methods

### Study Area

**FIGURE 1:**
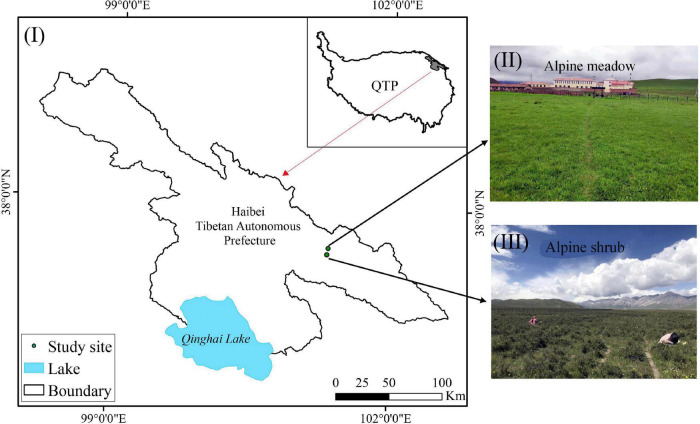
The location of study area **(I)**, alpine meadow **(II)**, and alpine shrub **(III)**.

This study was conducted at the Haibei National Field Research Station, Qinghai, China (37°37N, 101°19E), at an elevation of 3,200 m ASL on the northeastern QTP (Figure 1). This region has a plateau continental monsoon climate with an average annual temperature of −1.7°C and monthly mean temperatures ranging from −15.0°C in January to 10.1°C in July. The annual precipitation is 580 mm, of which 80% falls between May and September (the growing season), and the annual pan evaporation is approximately 1,191.4 mm. Two vegetation types were selected for this region: alpine shrub and alpine meadow (Figure 1). The alpine shrubs and alpine meadows were mainly dominated by *Potentilla fruticosa* and *Kobresia humilis*, respectively. Both vegetation types were close to the meteorological station at Haibei National Field Research Station. Seasonally frozen ground is well developed in this region, and the groundwater level is approximately 3–5 m (Dai et al., 2019b). For the alpine meadow, there was a thick mattic epipedon layer in the topsoil at a depth of 10–30 cm. The mattic epipedon has a relatively high water-holding capacity and low heat conductivity. The basic soil properties of the two vegetation types are presented in Table 1.

**TABLE 1:** Vegetation and soil characteristics at 0–40 cm across two vegetation types in the study area.

Vegetation type	Dominant species	Soil depth (cm)	Soil bulk density (g cm^–3^)	Soil organic matter (g kg^–1^)	Clay content (%)	Silt content (%)	Sand content (%)	Saturated water (%, v/v)	Field water (%,v/v)
Alpine meadow	*Kobresia humilis*	0–20	0.90	91.22	4.66	47.22	48.12	56.50	38.47
		20–40	1.06	60.97	7.58	60.54	31.88	53.38	34.15
Alpine shrub	*Potentilla fruticosa*	0–20	0.72	118.35	6.81	67.06	26.13	72.40	42.96
		20–40	0.98	75.65	6.23	60.17	33.60	63.17	43.44

### Experimental Design

Three research plots (25 m × 25 m) were selected for each vegetation type and were located on flat terrain where no runoff occurred. To explore the soil water content variation under different soil layers, we defined soil layers of 0–20, 40–60, and 80–100 cm to represent the surface horizon, middle soil layer, and deep soil layer, respectively (Yao et al., 2013). In this study, the mean surface soil water content (0–20 cm) was sampled at 5-, 10-, 15-, and 20-cm depths, the mean middle soil water content (40–60 cm) was sampled at 40- and 60-cm depths and the mean deep layer soil water content (80–100 cm) was sampled at 80- and 100-cm depths.

To investigate the soil moisture response to precipitation infiltration under different vegetation types, we measured the soil moisture content 1–2 days before precipitation events, 1–2 days after precipitation events, and before the next precipitation event. We selected four different precipitation events in 2016 to assess changes in the vertical soil water distribution following each event (Gao et al., 2014). A low precipitation event (2.6 mm) occurred on April 24, a medium precipitation event (16.5 mm) occurred on June 13 and 14, a high-precipitation event (37.4 mm) lasted from August 12 to 14, and an extremely high-precipitation event (68.1 mm) lasted from June 19 to 25.

### Soil Properties and Biomass Measurement

The volumetric soil moisture was measured from 2015 to 2016 using the soil moisture sensor for the two vegetation types at depths of 5, 10, 15, 20, 40, 60, 80, and 100 cm, with three sensors per depth. This probe continuously measures soil moisture and updates a data logger (CR10X, Campbell Scientific, Logan, UT, United States) every 30 min. To avoid the effect of soil properties on the accuracy of soil water content measured by a soil moisture sensor, we adopted a manual field measurement method to calibrate the soil water content. This was done using a cutting-ring sampler to obtain an undisturbed soil sample at the same depth around the buried soil moisture sensors each month, repeated four times at each depth. The manually measured soil moisture was analyzed using linear regression with the soil moisture measured using a soil moisture sensor to obtain the calibration coefficient. Meteorological data for the study period, which include precipitation, air temperature, and wind velocity, were obtained from an automatic meteorological station located less than 60 m from the study site. To examine the effects of soil properties on soil moisture, we collected soil samples at depths of 0–20 and 20–40 cm, with three replicates. Soil organic matter was determined using the Walkley–Black method (Nelson and Sommers, 1996), and the soil particle distribution was measured using a MasterSizer 2000 (Malvern Instruments Ltd., United Kingdom) to obtain the clay (<0.002 mm), silt (0.002–0.02 mm), and sand (0.02–2 mm) contents. The soil bulk density was calculated as the ratio of the oven-dried soil mass to the core volume, and saturated water and field water were obtained using the cutting-ring method (Dai et al., 2020a).

Furthermore, the aboveground biomass was also obtained monthly during the growing season (i.e., from May to September) in 2015–2016, with the aim of exploring the effect of vegetation coverage on soil moisture content. The aboveground biomass was obtained using the standard harvesting method in 10 randomly selected quadrats (25 cm × 25 cm) and then oven-dried at 65°C to a constant weight.

### Data Analysis

First, a one-way analysis of variance (ANOVA) was used to test the effects of vegetation type on soil moisture content, followed by Tukey’s HSD test to compare differences in the mean water content for different land uses and soil layers when the results of the ANOVA were significant at *p* < 0.05. Second, Pearson’s correlation analysis was used to examine the relationship between meteorological factors and soil moisture. All analyses were conducted using R version 3.4.3 (R Development Core Team, 2006).

## Results

### Precipitation Patterns

**FIGURE 2:**
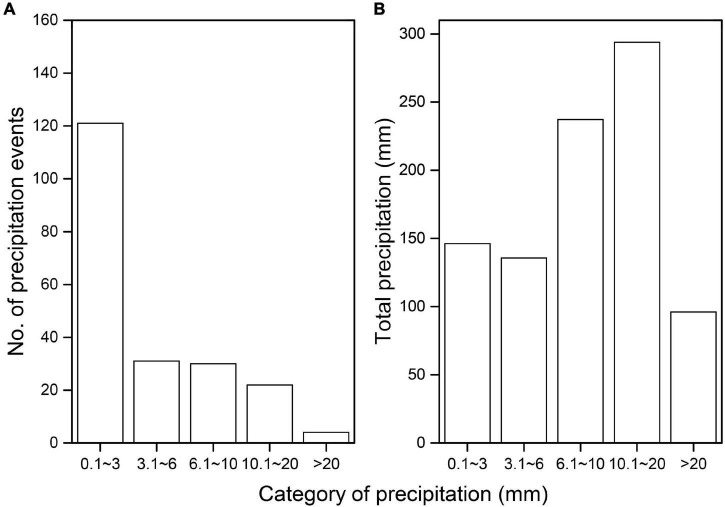
Number of precipitation event **(A)** and total precipitation **(B)** amount across different category precipitation.

**FIGURE 3:**
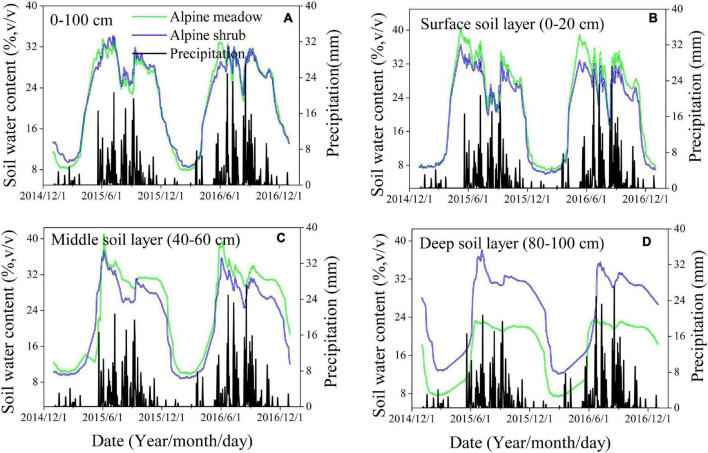
Daily variations of soil water content for 0–100 cm **(A)**, surface soil layer **(B)**, middle soil layer **(C)**, and deep soil layer **(D)** across two vegetation types.

From January 2015 to December 2016, 208 precipitation events were recorded, amounting to a cumulative total of 908.9 mm during the research period (Figure 2B). The minimum precipitation for any individual event within the 208 events was 0.1 mm and the maximum was 27.2 mm. The most prevalent precipitation event was a small precipitation event (<3.0 mm of precipitation), which accounted for almost 58% of all precipitation events (Figure 2A). Other precipitation event types, corresponding to 3.1–6 mm, 6.1, 10, and 10–20 mm precipitation, accounted for 15, 14, and 11% of all precipitation events, respectively (Figure 2A). Precipitation events >20 mm accounted for 2% of all the precipitation events (Figure 2A). Almost 86% of the precipitation occurred during the growing season (from May to September) when most of the greater precipitation (>3 mm) events occurred (Figure 3).

### Temporal Variations in Soil Moisture for the Two Vegetation Types

**FIGURE 4:**
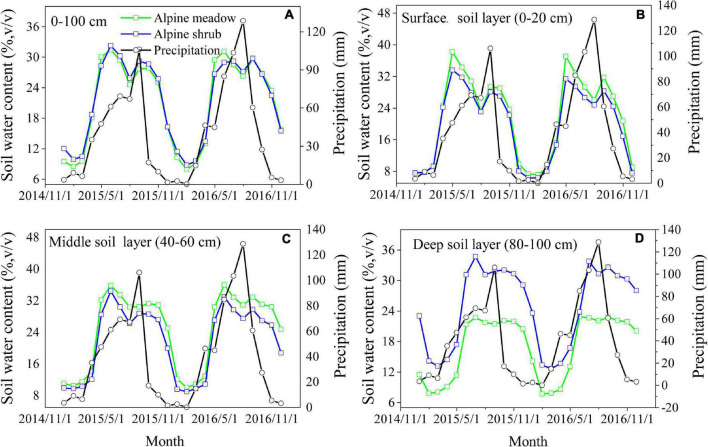
Monthly variations of soil water content for 0–100 cm **(A)**, surface soil layer **(B)**, middle soil layer **(C)**, and deep soil layer **(D)** across two vegetation types.

In general, the daily variations in 0–100-cm soil moisture for the two vegetation types were consistent with the temporal variations in precipitation, and soil moisture increased sharply after each precipitation event, particularly after a heavy precipitation event (Figure 3A). Surface soil moisture was more sensitive to precipitation events, whereas there was a clear lag in the moisture response for the deep soil layers (Figures 3B,D). The soil moisture content in the surface and middle layers was higher for alpine meadows than for alpine shrubs, whereas the soil moisture content in the deep soil layer was lower for alpine meadows than for alpine shrubs (Figures 3B,C). There was a clear seasonal pattern in soil moisture for the two vegetation types, which was characterized by two peaks for the upper soil layers in both 2015 and 2016 (Figure 4). The peaks for the surface soil water content occurred in May and September, with a much higher content in May than in September (Figure 4B). There was a single peak in the deeper soil layers for both vegetation types in July (Figure 4D).

### Comparisons Between the Vertical Soil Moisture Distributions for the Two Vegetation Types

**FIGURE 5:**
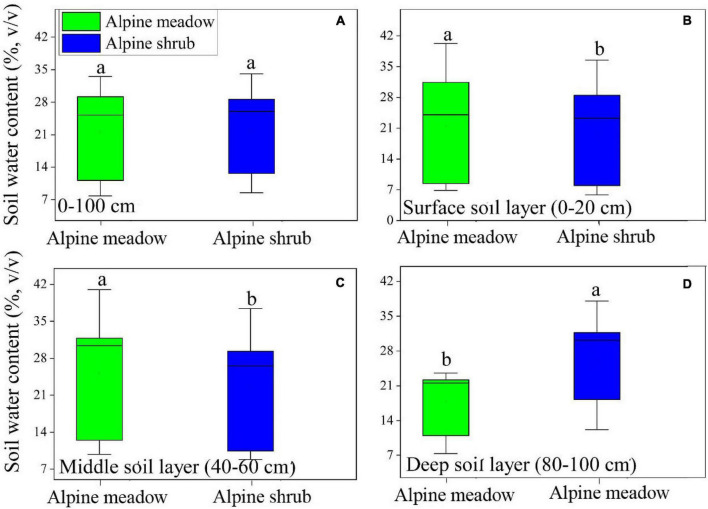
Comparison of soil water content for 0–100 cm **(A)**, surface soil layer **(B)**, middle soil layer **(C)**, and deep soil layer **(D)** across two vegetation types.

Significant differences were observed between the three soil layers for the two vegetation types (Figure 5). The soil moisture content was significantly (*p* < 0.05) higher for alpine meadows than for alpine shrubs in the surface and middle soil layers, whereas the deep soil moisture content of alpine shrubs was significantly higher than that of alpine meadows, which ultimately leads to no significant difference in 0–100 cm between the two vegetation types (Figure 5). There was an increasing trend in soil moisture from the surface to deep soil layers for alpine shrubs, whereas the soil water content for alpine meadows first increased and then decreased from the surface to deep soil layers, with maximum moisture occurring in the middle soil layer (Figure 5).

### Determining Factors of Soil Moisture Content for the Two Vegetation Types

**FIGURE 6:**
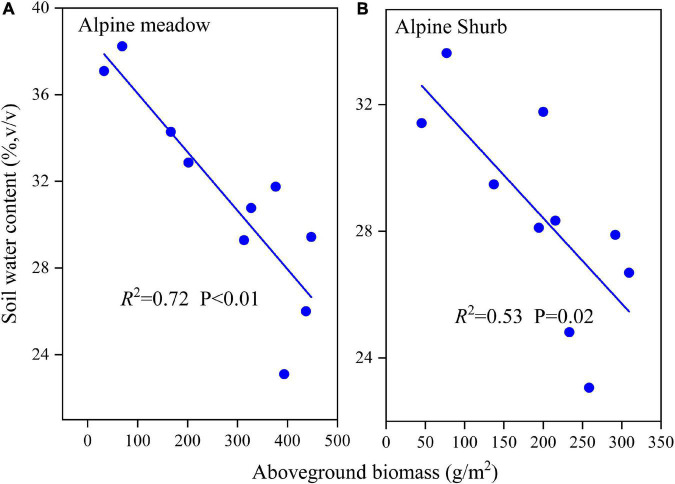
The relationship between aboveground biomass and soil water content for alpine meadow **(A)** and alpine shrub **(B)**.

Pearson’s correlation coefficient (r) was calculated between the monthly mean soil moisture in each soil layer and meteorological observations (Table 2). The results showed a significant positive correlation (*p* < 0.01) between soil moisture and precipitation for alpine shrubs for all soil layers, except for the 80–100-cm soil layer, whereas the average air temperature was significantly negatively correlated with soil moisture (Table 2). For alpine meadows, we observed a significant positive correlation (*p* < 0.01) between soil moisture and precipitation at all soil depths, whereas average air temperature only had a significant negative impact at depths of 0–80 cm (Table 2). In addition to these meteorological factors, aboveground biomass had a significant negative effect on the monthly average surface soil moisture content in both alpine meadows and alpine shrubs during the growing season (Figure 6).

**TABLE 2:** Pearson’s correlation between precipitation and soil moisture at different soil layers for alpine shrub and alpine meadow.

Correlation coefficient	Alpine shrub		Alpine meadow	
		
	PPT	T_ave_	PPT	T_ave_
5 cm SWC	0.71***	−0.86***	0.58***	−0.76***
10 cm SWC	0.68***	−0.86***	0.68***	−0.85***
15 cm SWC	0.67***	−0.84***	0.66***	−0.82***
20 cm SWC	0.65***	−0.81***	0.63***	−0.78***
40 cm SWC	0.63**	−0.76***	0.59**	−0.72***
60 cm SWC	0.60**	−0.68***	0.55**	−0.62**
80 cm SWC	0.46*	−0.44*	0.47*	−0.47*
100 cm SWC	0.34	−0.28	0.44*	−0.41

*SWC, soil water content; PPT, precipitation; T_ave_, average air temperature.*

*Indicates that the correlation is significant at p < 0.05.

**Indicates that the correlation is significant at p < 0.01.

***Indicates that the correlation is significant at p < 0.001.

### Response of Soil Moisture to Precipitation Patterns

**FIGURE 7:**
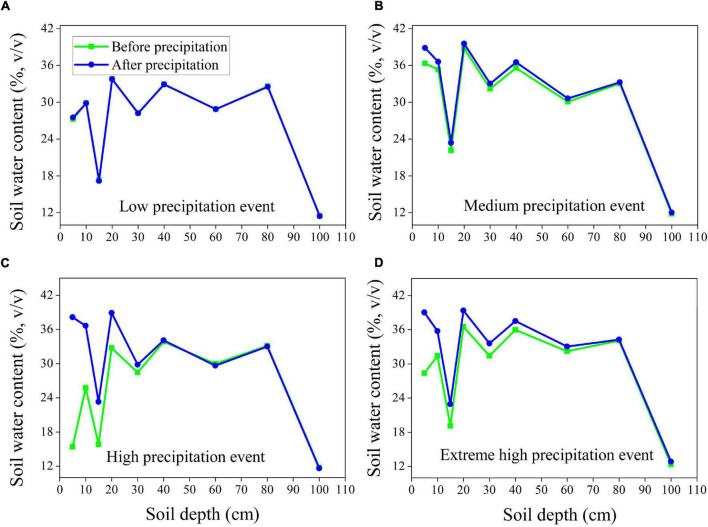
The depth of soil water infiltration of alpine meadow at low precipitation event **(A)**, medium precipitation event **(B)**, high-precipitation event **(C)** and extremely high-precipitation event **(D)**.

**FIGURE 8:**
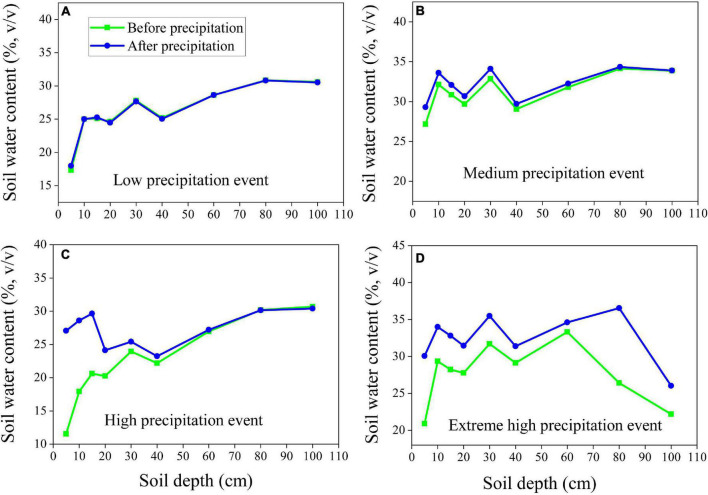
The depth of soil water infiltration of alpine shrub at low precipitation event **(A)**, medium precipitation event **(B)**, high-precipitation event **(C)** and extremely high-precipitation event **(D)**.

To analyze variations in soil moisture and relate them to different precipitation amounts, we examined the differences between soil moisture before and after precipitation for four precipitation events (Figure 7). We found that the low precipitation event (2.6 mm) had no effect on precipitation infiltration for either vegetation type (Figures 7A, 8A), whereas the medium precipitation event (16.5 mm) replenished the soil moisture content of the uppermost 15 cm for the soil under the alpine meadow (Figure 7B) and replenished the moisture at depths up to 40 cm for the soil under the alpine shrub (Figure 8B). When the precipitation amount was high (47.4 mm), the maximum infiltration depth was 60 cm for the alpine meadows (Figure 7C) and 80 cm for the alpine shrubs (Figure 8C). When the precipitation amount was extremely high (68.1 mm), the maximum infiltration depth was 60 cm for alpine meadows (Figure 7D) and >100 cm for alpine shrubs (Figure 8D).

## Discussion

### Temporal Variability and Controlling Factors for Soil Moisture for the Two Vegetation Types

Soil moisture variability is a key process in the hydrological cycle of terrestrial ecosystems and is affected by many factors such as soil properties, precipitation patterns, groundwater levels, and the characteristics of the overlying vegetation (Yang et al., 2012; Niu et al., 2015). In this study, the groundwater level was relatively shallow (3–5 m); therefore, precipitation was the primary controlling factor for soil moisture variability in the shallow and middle soil layers. For these layers, we found that the variations in soil moisture were consistent with the temporal variations in precipitation for both vegetation types (Figure 3). The soil moisture increased sharply after precipitation events, particularly following heavy precipitation events. This result concurs with the findings of the previous studies conducted in different precipitation regions, which found that higher soil water content is always accompanied by higher precipitation (Zhao et al., 2017; Mei et al., 2018). Precipitation patterns, therefore, determine the observed temporal variations in soil moisture, and the substantial seasonal variations resulting from the uneven distribution of precipitation. In our study region, almost 86% of the total annual precipitation falls during the growing season (Figure 3), which results in highly observed soil moisture levels which drive plant growth. However, the soil moisture during the non-growing season was relatively low owing to less precipitation, and most of the liquid water was frozen in the ground (Dai et al., 2019b). Precipitation in our study area was characterized by small events (<3.0 mm of precipitation), which accounted for almost 58% of all precipitation events, whereas events with more than 20 mm of precipitation accounted for only 2% of all precipitation events (Figure 2). We found that small precipitation events did not lead to an increase in soil moisture, and some cases, there was a slight reduction where evaporative demands were high (Figure 3). Our results suggest that the effect of a series of small rainfall events is not necessarily equivalent to the effect of the same total rainfall amount from a single large event, because the water from most small rainfall events quickly evaporates into the atmosphere instead of being absorbed by the plant. In contrast, larger rainfall events could immediately increase soil moisture, subsequently enhancing soil moisture storage and infiltration, which provides abundant nutrients for plants.

Furthermore, we found that the soil moisture content in the surface and middle soil layers was more sensitive to precipitation events than the deep soil moisture content. When the precipitation amounts reached their maximum, the surface soil moisture content also reached its maximum (Figure 3), whereas the soil moisture content for the deep soil layer displayed a smooth temporal change and exhibited a clear lag in response to precipitation (Figure 3D). Moreover, the same temporal variations in soil moisture were observed for the two vegetation types in the surface, middle, and deep soil layers; however, the vertical distribution of soil moisture was different for the two vegetation types. Therefore, we conclude that precipitation was the dominant factor that affects the temporal trends of soil moisture, whereas vegetation type was the dominant factor that affects the spatial distribution of soil moisture. These results are consistent with the previous findings by Yao et al. (2012). The effect of precipitation on soil moisture in alpine ecosystems is complex and is affected by dynamic processes such as the amount, intensity, and duration of precipitation (Niu et al., 2015). In addition to the sensitivity of soil moisture to precipitation, we found a significant negative correlation between soil moisture content and air temperature (Table 2). This agrees with the previous studies that demonstrated that temperature drives a decrease in soil moisture under high moisture conditions because soil moisture loss after a rainfall event is primarily driven by atmospheric demand or energy (Wang et al., 2011). We also observed that soil moisture content was affected by vegetation characteristics, such as vegetation biomass (Figure 6). The previous studies have found that increasing aboveground biomass leads to a decrease in soil moisture because canopy interception and root water consumption reduce rainfall recharge to the soil (Chen et al., 2008), and this was corroborated by our findings that soil moisture was negatively related to aboveground biomass during the growing season (Figure 6). In general, higher aboveground biomass leads to higher canopy coverage and leaf area index, which promotes the interception of precipitation and subsequent evapotranspiration of soil water, thereby reducing soil moisture (Zhang et al., 2020). Nevertheless, increased canopy coverage can impact the local soil moisture in arid regions because wind speed and temperature are decreased *via* shading, thereby reducing evaporation.

### Differences in Soil Moisture Content and Infiltration Processes for Two Vegetation Types

Infiltration processes are complex and affected by factors such as precipitation patterns, ground cover, soil characteristics, topography, and initial soil moisture (Gabarrón-Galeote et al., 2013; Wang et al., 2013). In this study, we found that low amounts of precipitation did not affect the soil moisture for the two vegetation types, possibly because moisture in the soil surface layer was rapidly lost through direct evaporation and transpiration, as no surface runoff occurred during the study period (Wang et al., 2012). The medium precipitation event affected only the uppermost 15 cm of the alpine meadow soil but reached the uppermost 40 cm of the alpine shrub soil. The deeper infiltration depth for alpine shrubs compared to alpine meadows may be related to the low bulk density and thus higher soil porosity for alpine shrubs (Table 1). In addition, alpine shrubs have a deeper root system that favors precipitation infiltration because root channels increase soil macroporosity, which leads to higher infiltration and preferential soil flow (Wu et al., 2017). Overall, the infiltration depth increased with precipitation amount for both alpine meadows and alpine shrubs, but the maximum infiltration depth of alpine shrubs was higher than that of alpine meadows under extreme high-precipitation events. This suggests that alpine shrubs might be less susceptible to surface runoff from increasing precipitation variability due to future climate change. Furthermore, alpine meadows have been experiencing rapid shrub encroachment on the QTP, which could favor precipitation infiltration and reduce the occurrence of surface runoff. It should be noted that, although a positive correlation between precipitation and infiltration depth has been widely reported in the previous studies (Yao et al., 2013), it remains difficult to quantify the relationship because of the differences in initial soil moisture, rainfall intensity, and vegetation cover among different precipitation events (Gao et al., 2014). Furthermore, only one measurement point for each soil depth before and after a precipitation event might be insufficient to draw a conclusion on infiltration depth; thus, adding more points should yield more accurate measurements of infiltration depth in future studies. In addition, seasonally frozen ground is well developed in this region, and the infiltration processes are greatly affected by the freeze-thaw cycle (Dai et al., 2019b). For instance, soil water infiltration was reduced during the soil freezing phase, whereas soil water infiltration was promoted during the soil thawing phase, which was confirmed after observing lower soil moisture during the non-growing season but higher moisture during the growing season (Figure 3). Further studies are needed for elucidating the relationship between the processes of soil moisture infiltration and soil freeze-thaw to attain a more comprehensive understanding of soil moisture infiltration processes.

Furthermore, we found that the surface moisture content of alpine meadows was higher than that of alpine shrubs (Figure 5). This difference may be attributable to differences in evapotranspiration due to the different root distributions and soil physical properties of the two vegetation types (Fu et al., 2003). The surface soil moisture content was higher in alpine meadows than in alpine shrubs, which may be explained by the following arguments. First, alpine shrubs have higher canopy heights than alpine meadows, which can intercept a greater proportion of incident precipitation *via* its thick branches and luxuriant foliage (Gao et al., 2014), as well as increased water evaporation, and reduced topsoil water content. A previous study found that the alpine shrub community canopy intercepts 11.7% of precipitation (Wang et al., 2005). Second, alpine shrubs have a deeper root system than alpine meadows, which leads to higher infiltration and preferential soil flow owing to higher soil macroporosity, which reduces surface soil water content but increases soil water content. Third, the alpine meadow has a thick mattic epipedon layer in the topsoil at 10–30 cm, which has a relatively higher water holding capacity, thus leading to higher surface soil moisture levels of alpine meadows (Dai et al., 2019a).

## Conclusion

Temporal variations in surface soil moisture for the two vegetation types were predominately controlled by temporal variations in precipitation and aboveground biomass, whereas there was a clear lag in the soil moisture response to precipitation events in deeper soil layers (80–100 cm). Spatial variability in soil moisture was explained by the differences between the two types of overlying vegetation. There were significant differences in the soil water content for the surface (0–20 cm), middle (40–60 cm), and deep (80–100 cm) soil layers in both alpine meadows and alpine shrubs. Soil moisture content was significantly positively correlated with precipitation, but the correlation coefficient decreased with soil depth. Air temperature and aboveground biomass were significantly negatively correlated with SM. Alpine shrubs had a deeper infiltration depth than alpine meadows under extreme high-precipitation events, which indicates that alpine shrubs might be less susceptible to surface runoff under these events. Furthermore, we found that the impact of a series of small rainfall events was not necessarily equivalent to the impact of the same total rainfall amount provided in a single large event.

## Data Availability Statement

The original contributions presented in the study are included in the article/supplementary material, further inquiries can be directed to the corresponding author.

## Author Contributions

LD and XG performed the research, analyzed the data, and wrote the manuscript. FZ, RF, and YD analyzed the data. GC conceived the study. All authors contributed to the article and approved the submitted version.

## Conflict of Interest

The authors declare that the research was conducted in the absence of any commercial or financial relationships that could be construed as a potential conflict of interest.

## Publisher’s Note

All claims expressed in this article are solely those of the authors and do not necessarily represent those of their affiliated organizations, or those of the publisher, the editors and the reviewers. Any product that may be evaluated in this article, or claim that may be made by its manufacturer, is not guaranteed or endorsed by the publisher.
